# Understanding the document bias in face matching

**DOI:** 10.1177/17470218211017902

**Published:** 2021-05-12

**Authors:** Xinran Feng, A. Mike Burton

**Affiliations:** Department of Psychology, University of York, York, UK

**Keywords:** Face matching, unfamiliar faces, bias effects

## Abstract

Matching unfamiliar faces is a well-studied task, apparently capturing important everyday decisions such as ID checks. In typical laboratory studies, participants make same/different judgements to pairs of faces, presented in isolation and without context. However, it has recently become clear that matching faces embedded in documents (e.g., passports and driving licences) induces a bias, resulting in elevated levels of “same person” responses. While practically important, it remains unclear whether this bias arises due to expectations induced by the ID cards or interference between textual information and faces. Here, we observe the same bias when faces are embedded in blank (i.e., non-authoritative) cards carrying basic personal information, but not when the same information is presented alongside a face without the card (Experiments 1 and 2). Cards bearing unreadable text (blurred or in an unfamiliar alphabet) do not induce the bias, but those bearing arbitrary (non-biographical) words do (Experiments 3 and 4). The results suggest a complex basis for the effect, relying on multiple factors which happen to converge in photo-ID.

## Introduction

A large body of psychological evidence shows that viewers are highly error-prone when matching images of unfamiliar faces (e.g., Bruce et al., 1999; Megreya & Burton, 2006). Despite this, photo-ID continues to be widely used in settings such as shops, workplaces, and airports, where staff need to match an ID document to the holder. In fact, viewers are generally unaware of their poor performance levels when matching unfamiliar faces (Zhou & Jenkins, 2020). However, most people are highly accurate at matching familiar faces (e.g., Bruce et al., 2001; Burton et al., 1999), and it appears that viewers over-generalise this ability to the faces of people they do not know (Ritchie et al., 2015).

Most research on face matching has employed simple image stimuli devoid of any context. However, similarly poor levels of performance are found in studies of viewers matching a photo to a live person in front of them (e.g., Kemp et al., 1997; Megreya & Burton, 2008; White et al., 2014). Furthermore, matching tasks involving images embedded in ID documents generally give rise to similar overall levels of accuracy as tasks using isolated face images (Bindemann & Sandford, 2011; Kramer et al., 2019; Meissner et al., 2013).

Despite the fact that accuracy in unfamiliar face matching appears consistently low across a number of settings, it has recently become clear that there are some key differences between different matching contexts. McCaffery and Burton (2016) directly compared the performance of viewers matching pairs of face images presented as isolated images or with one embedded in a passport. This simple manipulation did not affect overall accuracy but did significantly affect bias: participants were more likely to accept a pair of images as a “match” if one was embedded in a passport. Note that if such a bias were observed operationally, in real settings it would lead to a relaxation of criterion for accepting a match—that is, to more fraudulent presentations being accepted. McCaffery and Burton suggested that this bias effect seemed to be due to the presence of the passport frame and offered a number of possible reasons for this—including the apparent authority of the passport document, interference from task-irrelevant written information, or lower level visual context effects.

**Figure 1.**
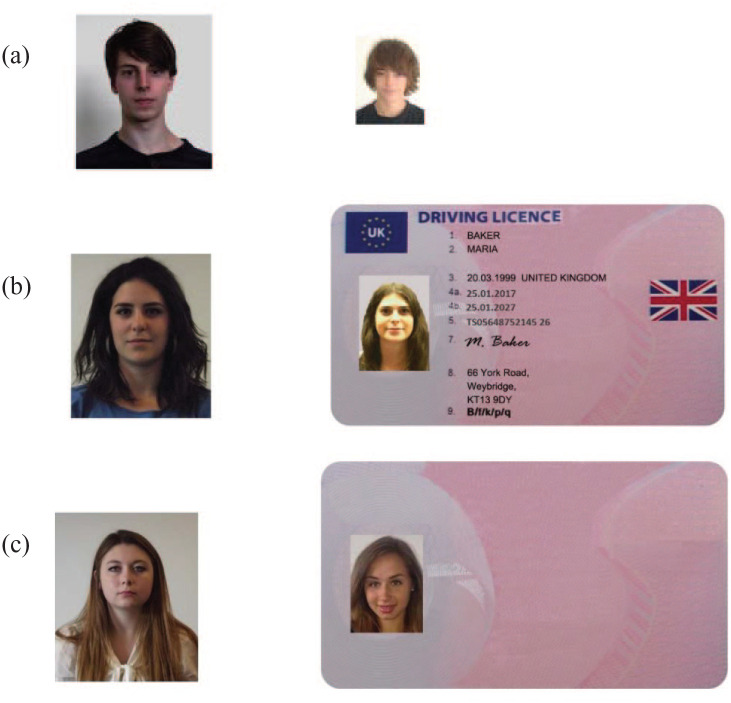
Examples of face stimuli used by Feng and Burton (2019). All face pairs were chosen from the Kent Face Matching Test (KFMT; Fysh & Bindemann, 2018). (a) Isolated faces. (b) One of the faces embedded in a driving licence frame. (c) A blank card derived from the driving licence. Pairs in this example all show different identities.

Feng and Burton (2019) replicated the “passport bias” and showed that it occurred in other documents, such as driving licences and student ID cards—even though the latter are considered to carry much lower authority than passports. However, they found that the bias was only present when the ID card carried personal information. So, for the examples shown in Figure 1, all three conditions gave rise to the same overall levels of face matching accuracy. However, in Condition (b), there was a significant bias such that viewers were more likely to make a “same person” response than in Condition (a) or (c).

In this article, we explore the nature of this effect, focussing on the task-irrelevant written information available to viewers in a face matching task. There are good reasons to hypothesise that the effect arises from interference between processing the faces and processing the biographical information carried on an ID card. Faces have been demonstrated to interfere with other perceptual tasks (Jenkins et al., 2003; Lavie et al., 2003), and textual labels such as names and occupations can interfere with face classification tasks (Young et al., 1986). However, the effect may reflect a more general contextual level of processing, involving viewers implicitly processing the entire ID setting. Priming tasks show clear semantic processing of faces (e.g., Boehm et al., 2006), and semantic contexts are known to influence face processing and recognition (Koji & Fernandes, 2010; Rainis, 2001; Schwartz & Yovel, 2016). So the information on card documents may interact with the faces when carrying out such matching tasks.

In addition to these perceptual/cognitive explanations, it is also possible that an ID card context carries with it an expectation that fraudulent use will be rare, biasing viewers towards a “same person” response. Although we have established that many different types of card elicit the bias (Feng & Burton, 2019), it remains possible that *any* ID card sets up an expectation that affects viewers’ responses. In a series of studies, we separate out the authoritative context of the card from the personal information it contains (Experiments 1 and 2). Having established that card context is critical to elicit the effect, we then manipulate the readability of biographical information by rendering it in a script unknown to the viewers or by blurring (Experiments 3 and 4). Taken together, the results point to multiple sources of the observed bias, depending on both card and linguistic contexts.

## Experiment 1

**Figure 2.**
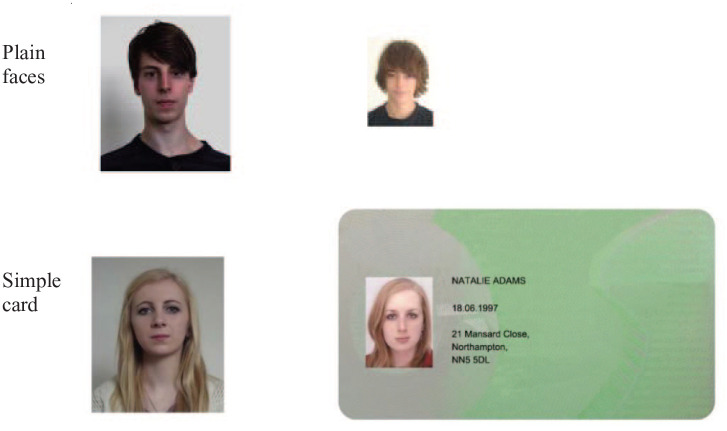
Example face pairs from two presentation conditions in Experiment 1. Each of these examples shows different identities.

In the first experiment, we examined the effect of minimal ID-like context on face matching. Card frames were created containing personal information (name, date of birth, and address), but no further cues about the purpose of the card. This simple layout, illustrated in Figure 2, preserves many of the features of a standard ID, but does not convey any information at all about its nature, that is, whether or not it carries official status. Comparison of the performance on standard isolated face matching and the minimal card context will establish a baseline ID effect, independently of expectations induced by specific contexts, such as passports, driving licences, or workplace IDs.

## Method

### Participants

Thirty-two students (27 females, aged from 18 to 24 years, mean age = 19.0 years) from the University of York participated for course credit. Sample size was based on previous experiments using similar procedures and stimuli (e.g., Bindemann et al., 2013; Feng & Burton, 2019). All participants reported normal or corrected-to-normal vision. Informed consent was provided prior to participation, and experimental procedures were approved by the ethics committee of the Psychology Department at the University of York.

### Stimuli

Sixty face pairs were randomly chosen from the Kent Face Matching Test (KFMT; Fysh & Bindemann, 2018). See Figure 2 for examples. These image pairs were taken several months apart and are presented at different sizes in the KFMT (see Fysh & Bindemann, 2018, for details). For each face pair, an item was created in which the smaller image was embedded in a card (Figure 2). This card contained the ostensible name, date of birth, and address of the holder, all fictitious and constructed for the purpose of the experiment.

### Design and procedure

In a within-subjects design, all participants completed two face matching blocks, as illustrated in Figure 2: plain faces and faces embedded in simple cards. There were 30 face pairs per block (15 matches and 15 mismatches), and pairs were counterbalanced across the experiment such that each appeared equally often in the plain and card-embedded conditions. Participants’ task on each trial was to indicate whether the face pair showed the same person or different people by pressing corresponding keys on a keyboard. Each face pair was displayed until a response was made. Order of block was counterbalanced across participants.

## Results and discussion

**Figure 3.**
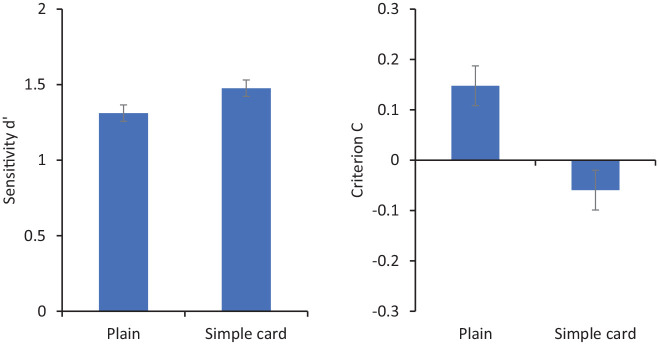
Sensitivity (*d′* on the left) and criterion (C on the right) for matching responses in Experiment 1. Error bars represent within-subjects standard error (Cousineau, 2005).

Figure 3 shows sensitivity (*d′*) and criterion (C) for matching decisions. For these purposes, “match” responses are coded as corresponding to *hits* when the two photos show the same person and as *false positives* when they show different people. Repeated-measures analysis of variance (ANOVA) showed no significant effect of condition on *d′*, *F*(1, 31) = 2.30, *p* = .14, $\eta_{p}^{2}=.07$, but a significant effect on C, *F*(1, 31) = 6.89, *p* = .013, $\eta_{p}^{2}=.18$. Mean criterion was more negative when participants saw faces embedded in card frames (*M* = −0.06) than when they saw faces alone (*M* = 0.15).

These results show a response bias such that participants viewing faces embedded in a simple card made more “same person” responses than they did when viewing plain, isolated faces. This echoes previous studies using more sophisticated ID documents such as passports and driving licences. The fact that this bias is elicited by so simple a presentation seems to suggest a rather minimal role for the perceived authority of a card. Of course, it may be that viewers implicitly attribute some official nature to the ID, but this card looks like neither of the two official documents routinely carried by our experimental participants—passports or driving licences.

This result raises the possibility that the biasing effect of card context is driven primarily by the text it contains. In the next study, we eliminate the card context altogether, preserving only the text. If the bias is observed in that condition, then it may need to be recast as a picture–word interference effect, rather than an effect tied to social use of ID, as has been previously suggested.

## Experiment 2

**Figure 4.**
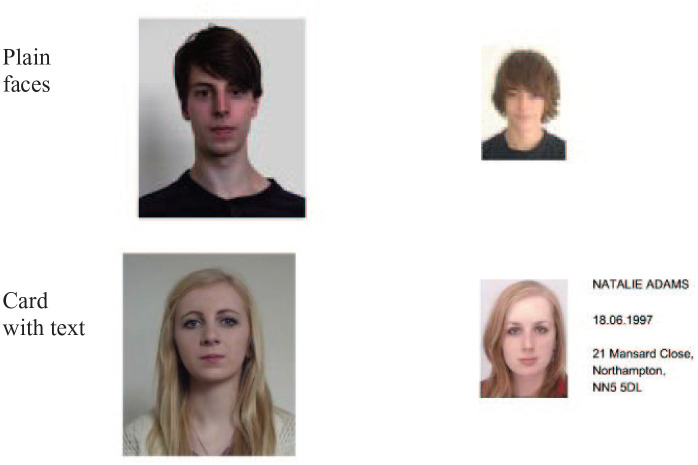
Face pairs from two presentation conditions in Experiment 2. Each of these examples shows different identities.

In this experiment, we compared isolated face matching to a presentation in which biographical information is presented alongside a face, but not within a card context. The same information was presented (name, date of birth, and address), in the same relative position as Experiment 1, but without any card context (see Figure 4). As in previous experiments, this information was task-irrelevant, and participants were simply asked to indicate whether two face images showed the same person or different people. If the bias, now reported across many ID contexts, is induced by fundamental picture–word interference, then we would expect to observe it in this presentation.

## Method

### Participants

Twenty-eight students (25 females, aged from 18 to 31 years, mean age = 20.9 years) from the University of York participated for course credit or a small payment. All reported normal or corrected-to-normal vision. Sample size was based on previous experiments using similar procedures and stimuli (e.g., Bindemann et al., 2013; Feng & Burton, 2019). Informed consent was provided prior to participation, and experimental procedures were approved by the ethics committee of the Psychology Department at the University of York.

### Stimuli

Face matching pairs were identical to those used in Experiment 1, that is, 60 face pairs from the KFMT (Fysh & Bindemann, 2018). Two conditions were constructed: plain faces and faces alongside text (see Figure 4). For the plain face condition, the original face pair images from the KFMT were used. For the text condition, the same text in the same relative position as in Experiment 1 was used, but without the card background.

### Design and procedure

The experiment employed a within-subjects design. Each participant performed two face matching blocks as illustrated in Figure 4 (plain faces, faces alongside text). They saw 30 face pairs (15 matches and 15 mismatches) in each block, and pairs were counterbalanced across the experiment such that each appeared equally often in the plain and “face alongside text” conditions. Participants’ task was to indicate whether the face pair they saw showed the same person or different people by pressing corresponding keys on a keyboard. Each face pair was displayed until a response was made. The order of the two blocks was counterbalanced across participants.

## Results and discussion

**Figure 5.**
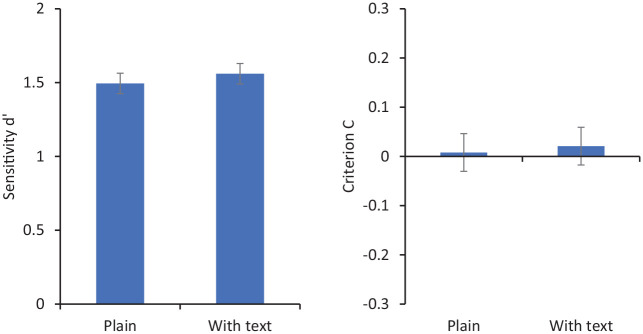
Sensitivity (*d′* on the left) and criterion (C on the right) for matching responses in Experiment 2. Error bars represent within-subjects standard error (Cousineau, 2005).

Figure 5 shows sensitivity (*d′*) and criterion (C) for matching decisions. Repeated-measures ANOVA showed no significant effect of presentation type on *d′*, *F*(1, 27) = 0.23, *p* = .64, $\eta_{p}^{2}=.01$, and no significant effect on C, *F*(1, 27) = 0.03, *p* = .87, $\eta_{p}^{2}<.01$.

This experiment showed no effect of adjacent biographical text on bias in face matching. This is despite the fact that exactly the same text embedded in a simple card layout does induce a bias (Experiment 1), a result consistent with previous research using realistic ID such as driving licences and passports. This result seems to rule out any simple explanation based entirely on textual interference on face matching. In the two experiments, the physical layout of the text and photos was identical, suggesting that the overall card context is critical to understanding this effect.

The first two experiments, combined, suggest that the effect of visual context on face matching relies on a complex combination of visual display characteristics, incorporating both biographical text and an implied ID card context. The effect of (biographical) text does not, therefore, appear to be automatic, but somehow facilitated by surrounding context. In the next two experiments, we explore this relationship further using ID cards with embedded text that is unreadable or irrelevant to participants due to it being rendered in an unfamiliar script (Experiment 3), blurred or semantically inappropriate to ID (Experiment 4).

## Experiment 3

**Figure 6.**
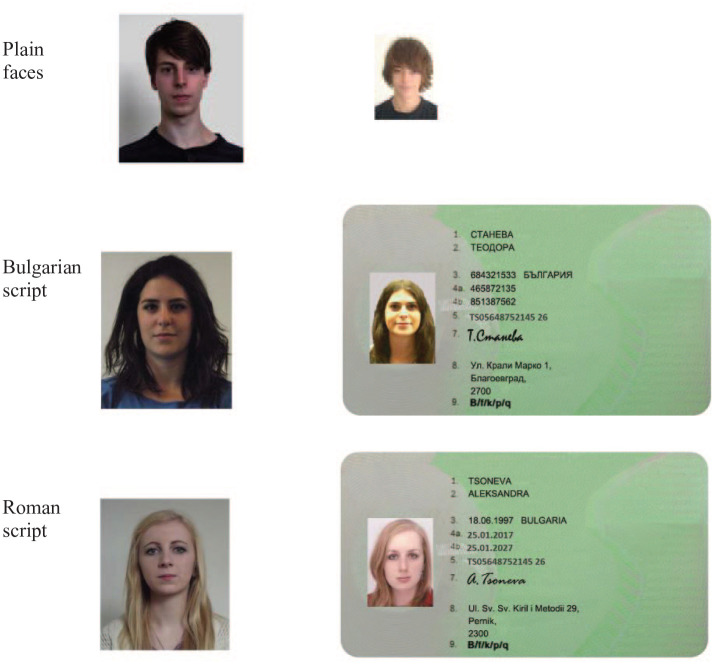
Face pairs from three presentation conditions in Experiment 3. Each of these examples shows different identities.

This experiment has a similar format to Experiments 1 and 2. Matching performance is compared for isolated faces and faces embedded in an ID card context. However, in Experiment 3, we present a card with the accompanying biographical text in Bulgarian—a language unfamiliar to the participants (see Figure 6). This information was presented either in the Bulgarian alphabet, rendering it literally unreadable by the participants, or transliterated into Roman script, rendering it readable, but mostly meaningless to participants. If the effect of context on face matching is carried mainly by the apparent fact that it is an ID card, irrespective of the content, then it should be observed for these cards in Bulgarian. However, if the source of the effect relies on processing the meaning of the biographical information, then it should not be observed at all in this experiment.

## Method

### Participants

Thirty students (27 females, aged from 18 to 26 years, mean age = 20.5 years) from the University of York participated for course credit or an amount of money. All reported normal or corrected-to-normal vision. A power analysis using GPower (Erdfelder et al., 1996) indicated that a sample of 30 participants would be needed to detect an effect of size $\eta_{p}^{2}=.2$, with 90% power using a within-subjects ANOVA and alpha at .05. Participants were pre-screened to ensure that they had no prior familiarity with Bulgarian or Russian language or culture. Informed consent was provided prior to participation, and experimental procedures were approved by the ethics committee of the Psychology Department at the University of York.

### Stimuli, design, and procedure

Face pairs were identical to Experiments 1 and 2. Biographical information for “Bulgarian” cards was created using the template of a UK Driving Licence, which has been shown to influence face matching in previous studies (see Figure 1). This included the card bearer’s name, address, and signature, along with various official designation numbers relating to the licence. The information was constructed with the help of a Bulgarian national and combined common forenames and surnames along with plausible addresses. For the Roman script versions, names and addresses were transliterated, for example, “Стоева Петя” to “Stoeva Petya” (Figure 6). While this renders them readable to participants, the names and addresses were nevertheless unfamiliar.

The design and procedure were similar to Experiments 1 and 2. Participants were asked to make face matching decisions (same/different) to pairs of faces. They completed three blocks of trials as illustrated in Figure 6: plain isolated faces, faces with cards in Bulgarian, and faces with cards in Bulgarian rendered in Roman script. Each block comprised 20 face pairs (half matching), and the order of blocks was counterbalanced across the experiment. Face pairs were also counterbalanced such that across the experiment, each pair occurred equally often in each condition.

## Results and discussion

**Figure 7.**
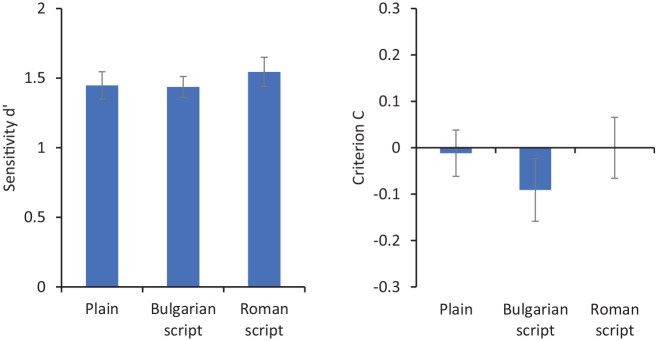
Sensitivity (*d′* on the left) and criterion (C on the right) for matching responses in Experiment 3. Error bars represent within-subjects standard error (Cousineau, 2005).

Figure 7 shows sensitivity (*d′*) and criterion (C) for matching decisions. Repeated-measures ANOVA showed no significant effect of presentation type on *d′*, *F*(2, 58) = 0.35, *p* = .71, $\eta_{p}^{2}=.01$, and no significant effect on C, *F*(2, 58) = 0.43, *p* = .65, $\eta_{p}^{2}<.01$.

This study shows that if people cannot read the information on cards, they are not biased in their responses. It seems, then, that the implied nature of the ID is not sufficient to produce an effect on face matching. Instead, it seems necessary that both card context and meaningful text are necessary to produce this effect. Note that the text rendered in Roman script was still insufficient to produce an effect, even though it was readable, but mostly not understandable by the participants. In the next experiment, we invert this relationship by including understandable but irrelevant text on cards (i.e., non-biographical English words).

## Experiment 4

The studies presented so far appear to demonstrate that to have an effect on face matching, it is necessary to present understandable information within a card context. Note that in all the experiments, and those in previous relevant research, cards and biographical information are task-irrelevant. This would suggest an automatic influence of proximate visual information. However, this is somewhat challenged by Experiment 2, showing no effect of adjacent text, outside a card context. In this final experiment, we introduced two new conditions. First, we constructed cards with readable, meaningful text, but this textual information was inappropriate to an ID card, simply comprising English nouns. Second, we presented a card with full, appropriate information, but in which the text was blurred. It was therefore clear that the card represented an ID card, such as shown in Figure 1b; however, it was not possible to read that information. Here, we wish to establish (1) whether only relevant text can influence matching and (2) whether cards with “implied” biographical information are sufficient to elicit an effect.

## Method

### Participants

Thirty-six students (29 females, aged from 18 to 32 years, mean age = 20.3 years) from the University of York participated for course credit or an amount of money. All reported normal or corrected-to-normal vision. A power analysis using GPower (Erdfelder et al., 1996) indicated that a sample of 30 participants would be needed to detect an effect of size $\eta_{2}^{p}=.2$ = .2, with 90% power using a within-subjects ANOVA and alpha at .05. Informed consent was provided prior to participation, and experimental procedures were approved by the ethics committee of the Psychology Department at the University of York.

### Stimuli, design, and procedure

**Figure 8.**
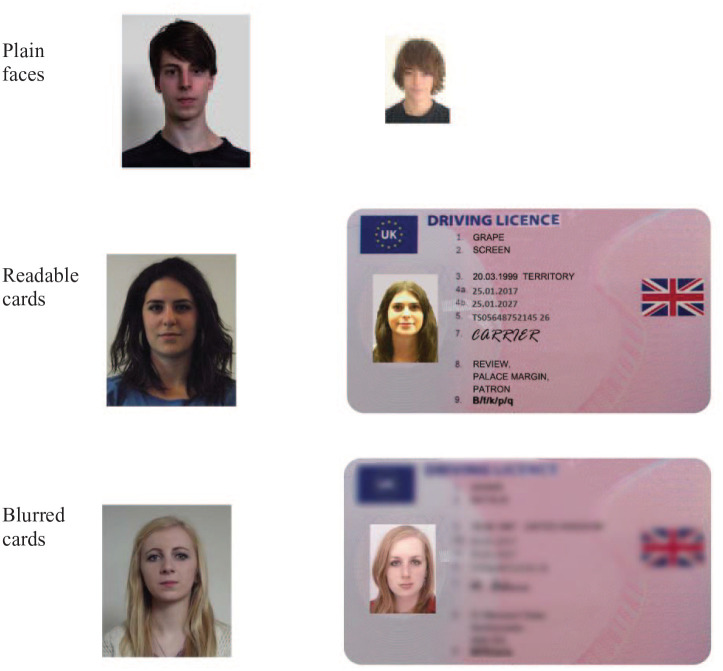
Face pairs from three presentation conditions in Experiment 4. Each of these examples shows “different” identities.

Face pairs were identical to Experiments 1–3. ID cards were designed using a UK Driving Licence template (Figure 8). For the “readable cards” condition, arbitrary nouns replaced the licence holder’s forename, surname, and address. For the “blurred” condition, plausible information was used for names and addresses (e.g., Figure 1b), but the textual part of the card was blurred to a level that preserved word shape but eliminated readability (see Figure 8).

Design and procedure were the same as Experiment 3. Participants were asked to make matching decisions (same/different) to pairs of faces. They completed three blocks of trials as illustrated in Figure 8: plain isolated faces, readable cards, and blurred cards. Each block comprised 20 face pairs (half matching), and the order of blocks was counterbalanced across the experiment. Face pairs were also counterbalanced such that across the experiment, each pair occurred equally often in each condition.

## Results and discussion

**Figure 9.**
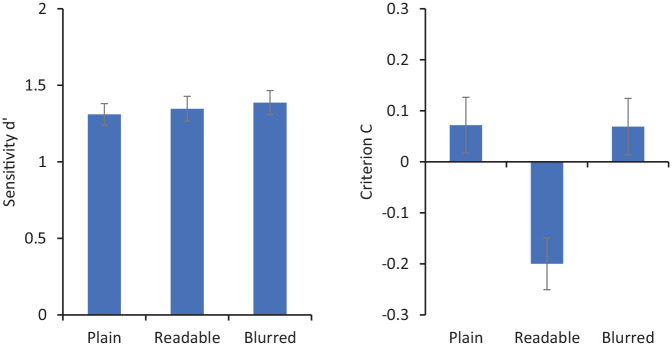
Sensitivity (*d′* on the left) and criterion (C on the right) for matching responses in Experiment 4. Error bars represent within-subjects standard error (Cousineau, 2005).

Figure 9 shows sensitivity (*d′*) and criterion (C) for matching decisions. Repeated-measures ANOVA showed no significant effect of presentation type on *d′*, *F*(2, 70) = 0.17, *p* = .85, $\eta_{p}^{2}<.01$, but a significant effect on C, *F*(2, 70) = 5.65, *p* = .005, $\eta_{p}^{2}=.14$. Pairwise comparisons were conducted using Tukey’s honest significant difference (HSD). Based on HSD = .225, the readable condition (*M* = −0.199) was significantly smaller than the plain condition (*M* = 0.072) and the blurred condition (*M* = 0.069). The plain and blurred conditions did not differ significantly.

These results show that participants made more “same person” responses to a face pair when one of the faces was embedded in a readable card, compared with a card in which they cannot see the information clearly or with plain isolated faces. This biasing effect with readable words replicates the findings with simple ID cards in Experiment 1 and those reported in previous studies with more formal ID (Feng & Burton, 2019; McCaffery & Burton, 2016). What differentiates this finding from previous studies is that the information on these cards is entirely irrelevant to the ID. Indeed, the arbitrary nouns used are somewhat bizarre in an ID context. Nevertheless, they appear to influence the face matching task. This is in interesting contrast to the blurred condition. These cards give every appearance of being legitimate ID, though the participants cannot read the details—which are task-irrelevant anyway. Under these conditions, no bias is observed by comparison to isolated faces.

### General discussion

In this series of experiments, viewers were consistently biased to make “same person” judgements to pairs of faces when one of them was embedded in an ID card containing readable information. In each experiment, the comparison of interest is between pairs of isolated faces and pairs in which one face is embedded in a document. We note that, in the studies reported here, the “baseline” bias (for the “plain” condition) is somewhat variable between experiments. In fact, this is also observed in previous studies using these KFMT faces (Feng & Burton, 2019; Fysh & Bindemann, 2018); for example, Fox and Bindemann (2020) report data relating visual acuity to performance with these faces, and while variance in acuity (within the normal range) does not predict matching accuracy, it does, on some occasions, predict bias. For this reason, we have reported bias in comparison with “plain” conditions throughout, rather than trying to capture any absolute theoretical comparison (e.g., to a zero bias) which is not characteristic of the test stimuli. As we noted in the Introduction, laboratory-based studies of matching tend to use isolated face stimuli only, and we have shown here that these normal experimental conditions can give rise to different patterns of bias when one of the faces is embedded in a document—patterns observed despite using the same stimulus items across conditions.

This bias to respond “same person” has been observed in previous studies and tentatively attributed to the apparent authority of official ID documents or to interference effects between face and text processing (Feng & Burton, 2019; McCaffery & Burton, 2016). However, the results presented here demonstrate that neither of these factors is sufficient to account for the effect. Adjacent text without a card context does not elicit the bias (Experiment 2), but the addition of a very simple card context does (Experiment 1). Furthermore, “interfering” text needs to be comprehensible but not semantically relevant to elicit this bias (Experiments 3 and 4).

In fact, it is hard to reconcile the observed matching bias with explanations based on interference from irrelevant text. In typical interference tasks, distractor items are designed to be response-congruent or incongruent. Under those conditions, the literature contains many examples of interference between face and text processing (e.g., Jenkins et al., 2003; Stenberg et al., 1998; Young et al., 1986). However, in our studies, “interfering” textual information was either consistent with an ID (Experiment 1) or irrelevant to it (Experiment 4). In conditions where text was clearly present, but unreadable (Experiments 3 and 4), no bias was observed. It therefore appears that face matching is somehow biased, in part, by the deployment of resources diverted into task-irrelevant reading, rather than by any semantic processing of the text.

Perhaps the most puzzling aspect of these results is the fact that the “same person” bias does not appear in Experiment 2, in which faces are presented alongside biographical text, but without an ID card context. This does suggest that the card frame sets up some expectation in the viewer, such that information on the card is then processed. This raises an unanswered question about the nature of the card contexts. We do not know whether these have their operation simply by acting to bring disparate information together as a single Gestalt (a perceptual explanation) or somehow induce an expectation in viewers based on the social use of ID card (a more social explanation). In future research, it will be important to investigate these possibilities—perhaps by embedding biographical information in enclosures that group information together but are not card-like.

The direction of the observed bias is itself important to consider. In all the reported experiments in which faces are embedded in ID cards, viewers tend to make more “same person” decisions than they do with pairs of isolated face images. It is interesting to note that this is usually the direction of bias observed in experiments which use more realistic tasks. For example, Kemp et al. (1997) showed that supermarket staff were poor at checking the photo-ID of their customers, and the majority of their errors were accepting mismatched ID. Similarly, White et al. (2014) asked working passport officers to verify the photo-ID of volunteers carrying true or “fraudulent” documents. Error rates were surprisingly high, and the majority of these were made in accepting false matches. It is possible that our experience of photo-ID sets up this context. For example, Robertson and Burton (2021) report that a decision about whether or not to sell someone alcohol is more strongly determined by an age check from an ID card than a face comparison to that ID. Kramer et al. (2019) point out that we are familiar with the notion that our friends’ photo-ID may not look very like them. Generalising this experience to novel faces may support the bias observed here, particularly when taken alongside an expectation that fraudulent ID use is likely to be rare. Future studies may be able to manipulate this expectation, perhaps by adjusting the frequency of match/mismatch trials (Bindemann et al., 2010; Papesh & Goldinger, 2014).

In conclusion, the “document bias,” while frequently demonstrated, resists simple explanation. Explanations relying solely on a card’s authority have been excluded, and we have now demonstrated that simple textual interference cannot explain the effect. Instead, it appears to rely on convergence of different stimulus characteristics—readable text, within a card-like frame. This is characteristic of a typical photo-ID, though it would seem speculative to characterise this specifically as an ID-based bias. In future experiments, it may be necessary to consider more elaborate contextual information to understand the bias—perhaps manipulating the use to which viewers believe the card will be put. Our results also serve as a reminder about the generalisation of simple effects. There is now a large literature on face matching which almost all employs isolated face images, and almost all makes appeal to the relevance of applied problems. It appears that there is a systematic difference between simple experimental face matching and real-world matching using documents.
